# Meaning in life and smartphone addiction among Chinese female college students: The mediating role of school adjustment and the moderating role of grade

**DOI:** 10.3389/fpsyg.2023.1092893

**Published:** 2023-02-01

**Authors:** Hao Zhao, Shameem Rafik-Galea, Mimi Fitriana, Tianjiao Song

**Affiliations:** ^1^School of Education, Shandong Women's University, Jinan, China; ^2^Faculty of Education, Languages, Psychology and Music, SEGI University, Kuala Lumpur, Malaysia; ^3^Faculty of Arts and Science, International University of Malaya-Wales, Kuala Lumpur, Malaysia

**Keywords:** meaning in life, smartphone addiction, school adjustment, Chinese female college students, mediating effect, grade

## Abstract

**Background:**

The fact that female college students are more addicted to smartphones than male college students has raised public concerns. However, previous studies have rarely explored the mechanism of female college students’ smartphone addiction. Previous studies have shown that identity may affect the formation process of female college students’ smartphone addiction, and the identity of female college students in different grades may be different. Nonetheless, few studies have explored the grade differences in the formation process of female college students’ smartphone addiction.

**Methods:**

The present study examined the relationship between meaning in life, school adjustment, and smartphone addiction among Chinese female college students using a moderated mediation model in which school adjustment played a mediating role and grade played a moderating role. A total of 1,076 Chinese female college students (Age: 19.83 ± 1.11; 369 freshmen, 379 sophomores, and 328 juniors) completed an online questionnaire regarding meaning in life, school adjustment, and smartphone addiction.

**Results:**

(1) School adjustment mediated the relationship between meaning in life and smartphone addiction. (2) School adjustment had a partial mediating effect between meaning in life and smartphone addiction for female freshmen and sophomores, but it did not exist among female juniors. (3) The influence of school adjustment on female sophomores’ smartphone addiction was significantly stronger than that of female freshmen.

**Conclusion:**

The findings of this study advance our understanding of the potential impacts of meaning in life on smartphone addiction and provide a grade perspective for targeted prevention or intervention with female college students’ smartphone addiction.

## 1. Introduction

Smartphones have become daily necessities for modern people because of their convenience, interaction, and portability. According to the latest Statistical Report on China’s Internet Development, as of June 2022, there are 1.051 billion Internet users in China, and up to 99.6% of them use smartphones to surf the Internet (China Internet Network Information Center, 2022). Appropriate use of smartphones can satisfy people’s entertainment, study, social interaction, and shopping needs. However, excessive use of smartphones can easily lead to smartphone addiction (Ahn and Jung, 2016). Smartphone addiction is defined as “a condition where the use of smartphone has fulfilled a deep need (dependency, habitual, and addictive behavior) to the extent that the individual has difficulty conducting basic activities of daily life without the concurrent use of a smartphone, and as such caused neglect of other aspects of one’s life” (Sunday et al., 2021, p. 2). Smartphone addiction harms people’s physical and mental health. Smartphone addiction can cause headaches, vision loss, daytime sleepiness, scapular dyskinesia, cramps in the fingers, stiff finger joints, psoriatic arthritis, and pain in the upper back, neck, shoulders, and wrists (Shan et al., 2013; İNal et al., 2015; Akodu et al., 2018; Hughes, 2018; Megna et al., 2018; Mustafaoglu et al., 2021). Previous studies have revealed that smartphone addiction makes it difficult for college students to concentrate in class (Dietz and Henrich, 2014), leading to a decrease in their life happiness (Volkmer and Lermer, 2018; Kozan et al., 2019; Satici and Deniz, 2020). Serious smartphone addiction can lead to social anxiety (Konan et al., 2018), personality disorder (Zhang et al., 2018a,b), and even suicidal tendencies (Wang J. J., 2017; Wang X., 2017; Ismail et al., 2020; Lee et al., 2020) in college students. Regarding gender differences in smartphone addiction, many studies have consistently reported that female college students have a higher risk of smartphone addiction than male college students (Mok et al., 2014; Roberts et al., 2014; Zhu and Liu, 2017; Nayak, 2018; Yi et al., 2020; He and Xia, 2021).

Recent studies have verified that meaning in life is an important influencing factor of college students’ smartphone addiction (Çevik et al., 2020; Hu et al., 2022). Some scholars have found that meaning in life is closely related to freshmen’s school adjustment or a certain factor (e.g., emotional adjustment) of college students’ school adjustment (Steger et al., 2011; Li et al., 2022). Moreover, plenty of previous studies have indicated that a certain factor of school adjustment, such as emotional adjustment, learning adjustment, and interpersonal adjustment, is closely related to college students’ smartphone addiction (Aker et al., 2017; Boumosleh and Jaalouk, 2017; Kim et al., 2017; Bian et al., 2018; Li et al., 2021; Zhang et al., 2022). However, little is known about the role of school adjustment between meaning in life and college students’ smartphone addiction.

College students of different grades need to face different external environmental challenges (e.g., freshmen need to adapt to the university quickly.) so as to perform college students’ duties well. Through the interaction between internal and external factors, different grades of college students may form different identities (Li and Liu, 2010). Under the influence of different identities, individuals show different adaptive social behaviors (Oyserman, 2009a,b). When college students form a healthy identity, they may experience a high level of meaning in life, which can promote the development of their social adaptability (Steger et al., 2009; Li and Liu, 2010), thus reducing their risk of addictive behaviors (Ryan and Deci, 2017). On the contrary, if college students do not form a healthy identity, they may have an identity crisis, and they may experience a low level of meaning in life, which easily leads to social maladjustment (Steger et al., 2009; Li and Liu, 2010), further increasing their risk of addictive behavior (Settley, 2020). However, little is known about the grade difference of the mechanism of college students’ meaning in life on smartphone addiction.

### 1.1. The relationship between meaning in life, school adjustment, and smartphone addiction

Smartphone addiction is a new behavioral addiction. Smartphone addiction, also known as “smartphone use disorder” or “problematic use of smartphone,” has the following characteristics: (a) smartphone use is out of control, such as using smartphones too frequently or being unable to control the use of smartphones on important occasions (Park and Lee, 2011; Lin et al., 2014); (b) psychological dependence on smartphones, paying too much attention to smartphones (Montag and Reuter, 2017); and (c) negative effects on individuals’ interpersonal communication, study, work, physical health, and mental health (Lin et al., 2014, 2016). Consequently, given the above characteristics of smartphone addiction, it is crucial to explore the mechanism of smartphone addiction among college students, especially female college students.

Meaning therapy theory of Frankl (1963) suggests that the fundamental drive of human existence is to constantly seek the meaning and purpose of life to satisfy psychological needs, and the meaning in life is the basic element for an individual to experience happiness in life. Meaning in life refers to “the sense made of, and significance felt regarding, the nature of one’s being and existence” (Steger et al., 2006, p. 81). Steger and Frazier (2005) indicated that the stronger an individual’s meaning in life, the lower the possibility of addictive behaviors. People with low levels of meaning in life lack the motivation and goal to pursue the meaning of life, so it is easy for them to fill the gaps in their lives with some addictive behaviors (Thege et al., 2013; Eryilmaz, 2014; Zhang et al., 2015; Yao et al., 2016; Çevik et al., 2020; Zhao et al., 2020; Hu et al., 2022). Recent studies have provided empirical evidence for the relationship between meaning in life and smartphone addiction; that is, meaning in life was negatively related to college students’ smartphone addiction (Çevik et al., 2020; Hu et al., 2022).

School adjustment is a very important development task in the process of students’ growth, which is closely related to the meaning in life and smartphone addiction. School adjustment is defined as the process of happily participating in various school activities and achieving academic achievements in the school context (Ladd et al., 1997). As for the structural components of school adjustment, most scholars agree that school adjustment should include campus life adjustment, learning adjustment, emotional adjustment, and interpersonal adjustment (Zeng, 2009). Meaning in life theory suggests that a higher level of meaning in life promotes optimal adjustment across domains of functioning, including physical health, well-being, and academic achievement (Roepke et al., 2014; Glaw et al., 2017; Hooker et al., 2018). This notion has been supported by empirical studies among adolescents, young adults, and elderly people (Kleiman et al., 2013; Henry et al., 2014; Czekierda et al., 2017, 2019). Since college students spend most of their time in universities, school adjustment is a very important part of college students’ social adjustment. Previous studies have examined the relationship between meaning in life and a certain factor of school adjustment among college students. For example, one previous study has indicated that undergraduate students whose meaning in life is threatened experienced greater stress than those whose meaning in life is intact (Park and Baumeister, 2016). Meaning in life is also closely related to emotional adjustment. Some scholars have verified that meaning in life has a positive effect on college students’ life satisfaction (Steger et al., 2011; Lin, 2020) and well-being (McMahan and Renken, 2011). Besides, meaning in life is negatively related to college students’ depression and anxiety (Lin, 2020). However, there are few studies on the direct relationship between meaning in life and college students’ school adjustment. Up until now, only Li et al. (2022) conducted a three-wave longitudinal study among freshmen to explore the relationship between meaning in life and school adjustment, and the results showed that meaning in life was positively related to freshmen’s school adjustment. Thus, future research needs to explore the direct relationship between meaning in life and school adjustment on the basis of expanding the sampling range of college students.

Self-determination theory proposes that after an individual’s basic psychological needs are satisfied, his or her social adjustment will be better, and then his or her risk of addiction will be lower (Deci and Ryan, 2000; Duchesne et al., 2016; Ryan and Deci, 2017). If individuals cannot satisfy their psychological needs in social situations, they are prone to maladjustment, and maladjustment is likely to lead them to seek substitutes and compensation in other fields (Deci and Ryan, 2000; Kuzucu and Şimşek, 2013), which easily leads to addictive behaviors (Verstuyf et al., 2013; Li et al., 2016; Settley, 2020). Smartphones have the following functional characteristics: (a) collectivity of functions (Kwon et al., 2013a,b); (b) personalization and customization of content (Kim, 2013; Lin et al., 2014; Mok et al., 2014); (c) accessibility (Wu et al., 2013; Ahn and Jung, 2016); and (d) convenience (Wu et al., 2013; Ahn and Jung, 2016). Since smartphones have the above functional characteristics, it is easy to obtain alternative satisfaction or compensation by using smartphones when individuals are prone to maladjustment. However, excessive use of smartphones easily leads to addiction (Ahn and Jung, 2016). Some empirical studies support the notion of self-determination theory. For example, with regard to the relationship between emotional adjustment and college students’ smartphone addiction, some studies have reported that depression (Aker et al., 2017; Boumosleh and Jaalouk, 2017; Kim et al., 2017), anxiety (Aker et al., 2017; Boumosleh and Jaalouk, 2017; Kim et al., 2017), and loneliness (Kim et al., 2017; Alinejad et al., 2022) are positively related to college students’ smartphone addiction. Moreover, one empirical research confirmed that alexithymia not only directly affected college students’ smartphone addiction, but also indirectly affected their smartphone addiction through depression, anxiety, and stress (Gao et al., 2018). As for the relationship between learning adjustment and smartphone addiction, scholars verified that learning boredom has a positive effect on the smartphone addiction of college students (Bian et al., 2018). Moreover, a recent study has revealed that the higher the perceived academic pressure of college students, the more serious their smartphone addiction is (Zhang et al., 2022). Interpersonal adjustment is also closely associated with college students’ smartphone addiction. Previous studies have consistently verified that interpersonal adjustment is negatively related to smartphone addiction; that is, the better the interpersonal adjustment of college students, the lower their risk of smartphone addiction (Zhang Y. L. et al., 2017; Zhang Y. et al., 2017; Zhang et al., 2018a,b; Li et al., 2021). Summarizing the above studies, these studies mainly explored the relationship between a certain factor of school adjustment and smartphone addiction, but there was a lack of direct research on the relationship between school adjustment and smartphone addiction.

In summary, although previous studies have examined the relationship between college students’ meaning in life and freshmen’s school adjustment or a certain factor of college students’ school adjustment and the relationship between a certain factor of school adjustment and college students’ smartphone addiction, there is a lack of empirical study among female college students to explore: (a) the relationship between meaning in life and school adjustment; (b) the relationship between school adjustment and smartphone addiction; and (c) the mediating role of school adjustment in the relationship between meaning in life and smartphone addiction.

### 1.2. Grade differences

Grade differences in psychology and behavior are a very important field of psychological research. Some studies have examined the grade differences in Chinese college students’ smartphone addiction (Xie and Wang 2015; Jiang and Li, 2020). Specifically, Chinese college students’ smartphone addiction shows a trend of first increasing (from freshman year to sophomore year), then decreasing (from sophomore year to junior year), and then rising (from junior year to senior year), among which sophomores’ smartphone addiction is the most serious, followed by seniors, and freshmen and juniors have the lowest level of smartphone addiction (Xie and Wang 2015; Jiang and Li, 2020). As for the grade differences in meaning in life, some recent studies found that freshmen had the highest level of meaning in life, followed by juniors and seniors, and sophomores had the lowest level of meaning in life (Zhang and Zhou, 2020; Xie, 2021). Grade differences also exist in Chinese college students’ school adjustment. Scholars found that the school adjustment of freshmen and juniors is significantly stronger than that of sophomores (Pang and Wu, 2012). One empirical study compared the differences in college students’ school adjustment among four grades and confirmed that the school adjustment of sophomores is significantly lower than that of the other three grades (Chao, 2008). Besides, one study further revealed that the scores of freshmen and juniors are significantly higher than those of sophomores in interpersonal relationships, career adjustment, emotional adjustment, and self-adjustment (Meng, 2014). To sum up the above studies, grade differences in meaning in life, school adjustment and smartphone addiction among college students are as follows:

- Meaning in life: freshmen > juniors, seniors > sophomores.
- School adjustment: freshmen, juniors, seniors > sophomores.
- Smartphone addiction: sophomores > seniors > freshmen, juniors.

College stage is an important period for college students to complete their identity as “college students.” Identity can be conceptualized as a way to understand some parts or aspects of self-concept (Oyserman and Markus, 1998; Hogg, 2003). Identity-based motivation theory proposes that people will behave in a way consistent with the identity initiated in a certain situation. People adopt certain behaviors or do not adopt certain behaviors, which are influenced by the identity perception initiated in a certain situation (Oyserman, 2009a,b). It needs to be emphasized that this kind of identity initiated by a certain situation is not always stable, and the formation of identity is influenced by the degree to which possible selves conforms to the external environment requirements (Bruner, 1990, 1995; Li and Liu, 2010; Crocetti et al., 2012). Possible selves are the individual’s self-relevant expectations for the future, which can predict and inspire people’s actions (Oyserman and Markus, 1990). When possible selves conforms to the external environment requirements, individuals will form a healthy identity, which will help to establish the meaning and purpose of life, and then make individuals show good social adjustment (Marcia, 1966; Erikson, 1968, 1975; Steger et al., 2009). When possible selves conflicts with the external environmental requirements, individuals may have identity crisis, which will lead to the loss of individual’s meaning in life, and then lead to a series of negative consequences such as social maladjustment and lack of sense of belonging and responsibility (Marcia, 1966; Erikson, 1968, 1975; Steger et al., 2009; Li and Liu, 2010). College students have to face different challenges in their 3 or 4 years of college life. Freshmen who have just entered the university need to adapt to university life quickly (Lu, 2015; Zhang Y. L. et al., 2017; Zhang Y. et al., 2017). Sophomores may easily fall into the “sophomore slump” due to various internal and external factors (Gahagan and Stuart Hunter, 2008; Kennedy and Upcraft, 2010; Wang and Kennedy-Phillips, 2013; Webb and Cotton, 2018). Besides, juniors and seniors need to face the pressure of further education and employment (Lu, 2015; Zhang Y. L. et al., 2017; Zhang Y. et al., 2017). Besides being influenced by individual factors, the development of individual’s possible selves is also influenced by the social environment in which they live (Bruner, 1990, 1995; Crocetti et al., 2012). Consequently, facing the above-mentioned “challenges” of different grades, the possible selves of college students in different grades may be different. Combining the meaning therapy theory, the meaning in life theory, the self-determination theory, and the identity-based motivation theory, we can understand that the degree of conformity between college students’ possible selves and the school environment requirements in different grades may lead to different results of their identity (healthy identity or identity crisis), and then make college students in different grades have different levels of meaning in life. Different levels of meaning in life of college students in different grades may have different direct effects on their smartphone addiction. Moreover, in different grades, different levels of meaning in life of college students may also have different effects on college students’ school adjustment, and then lead to different levels of smartphone addiction. As mentioned above, previous studies have explored the grade differences of college students’ meaning in life, school adjustment and smartphone addiction. However, previous studies have seldom explored whether there are grade differences in the relationship between meaning in life, school adjustment, and smartphone addiction.

### 1.3. The present study

**Figure 1:**
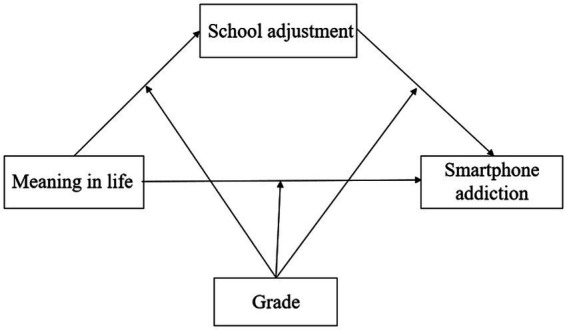
Conceptual model.

The present study proposed a moderated mediation model in which school adjustment played a mediating role in the relationship between meaning in life and smartphone addiction and grade played a moderating role in the relationship between meaning in life, school adjustment, and smartphone addiction. Specifically, combining the meaning therapy theory, the meaning in life theory, and the self-determination theory, this study proposed the hypothesis 1: School adjustment plays a mediating role between meaning in life and smartphone addiction among female college students. Based on the theoretical framework of hypothesis 1, this study combined with the identity-based motivation theory to further propose the hypothesis 2: The relationship between meaning in life, school adjustment, and smartphone addiction among female college students is moderated by grade. Figure 1 illustrates the conceptual model of the present study.

## 2. Materials and methods

### 2.1. Participants and procedure

The present study was approved by the Research Ethics Committee of Shandong Women’s University. The female college students from 50 classes at five universities in Shandong Province, China, were selected as participants by cluster random sampling in this study. Given the COVID-19 pandemic, the researcher distributed the online informed consent form and online questionnaire to the investigated classes through the Wen Juan Xing platform. First, all participants read the online informed consent form, which included a brief description of the purpose, significance, anonymity, and confidentiality of the questionnaire survey. Then, all participants signed the online informed consent form. Thirty-two participants were excluded for inaccurately completing the measures. Thus, the final sample consisted of 1,076 female college students, including 369 freshmen (34.29%), 379 sophomores (35.22%), and 328 juniors (30.48%). The average age of the subjects was 19.83 (SD = 1.11), and their ages ranged from 18 to 22.

### 2.2. Measures

#### 2.2.1. Meaning in life

Meaning in life was measured using the Chinese version of the Meaning in Life Scale (Liu and Gan, 2010). The scale includes two subscales: presence of meaning (e.g., “I know what can make my life meaningful”) and searching for meaning (e.g., “I am looking for the meaning of my life”), with nine items in total. Participants responded on a 7-point scale ranging from 1 (strongly disagree) to 7 (strongly agree). The higher the total score, the higher the level of meaning in life. The Cronbach’s α coefficient of the total scale in the present study was 0.86. The Cronbach’s α coefficients of the subscales of presence of meaning and searching for meaning were 0.84 and 0.85, respectively. The results of the CFA of the total scale showed that *χ*^2^/*df* = 1.768, *RMSEA* = 0.051 (90% CI: 0.038 to 0.069), *TLI* = 0.972, *CFI* = 0.975, *SRMR =* 0.038.

#### 2.2.2. School adjustment

Four subscales in the Chinese College Students Adjustment Scale (Fang et al., 2005), namely, campus life adjustment (e.g., “In everyday life, there are always things that interest me”), learning adjustment (e.g., “I have a high enthusiasm for my present study”), emotional adjustment (e.g., “I often adjust my emotional state by diverting my attention”), and interpersonal adjustment (e.g., “When I want to chat, I can always find someone to chat with”), were used to measure school adjustment. The four subscales have a total of 38 items. Participants responded on a 6-point scale ranging from 1 (disagree) to 6 (agree). A higher total score indicates better school adjustment. The Cronbach’s α coefficient of the total scale in the present study was 0.89. The Cronbach’s α coefficients of the subscales of campus life adjustment, learning adjustment, emotional adjustment, and interpersonal adjustment were 0.87, 0.87, 0.86, and 0.88, respectively. The results of the CFA of the total scale showed that *χ^2^*/*df* = 1.817, *RMSEA* = 0.062 (90% CI: 0.041 to 0.083), *TLI* = 0.968, *CFI* = 0.971, *SRMR =* 0.035.

#### 2.2.3. Smartphone addiction

Smartphone addiction was assessed using the Smartphone Addiction Scale - Short Version (SAS-SV) compiled by Kwon et al. (2013b), with 10 items in total (e.g., “Having a hard time concentrating in class, while doing assignments, or while working due to smartphone use”). Participants responded on a 6-point scale ranging from 1 (strongly disagree) to 6 (strongly agree). The higher the total score, the higher the level of smartphone addiction. The Cronbach’s α coefficient of this scale was 0.89 in the present study. The results of the CFA of the scale showed that *χ^2^*/*df* = 2.012, *RMSEA* = 0.059 (90% CI: 0.029 to 0.073), *TLI* = 0.973, *CFI* = 0.978, *SRMR =* 0.043.

### 2.3. Data analysis

Data were analyzed by using SPSS 26.0 and Mplus7.11 in the present study. The data analysis followed four steps:

First, common method bias analysis was conducted by SPSS 26.0. Specifically, this study used Harman’s One-Factor Test for factor analysis (unrotated exploratory factor analysis) for all items (Podsakoff et al., 2003). Among all factors with eigenvalues greater than 1 obtained after factor analysis, if the variance explained by the first factor is less than 40%, it indicates that there is no common method bias in the study (Podsakoff et al., 2003).

Second, descriptive statistics and Pearson correlation analysis were conducted by SPSS 26.0.

Third, grade was coded with dummy variables and set as the control variable by using SPSS 26.0, and then Mplus7.11 was used to test the mediation model.

Fourth, Mplus7.11 was used to conduct multiple group analysis to test the moderated mediation model. Specifically, this study tested the measurement invariance according to the following four steps: (a) Fit the measurement model separately for each group. (b) Model 1: Configural invariance. (c) Model 2: Metric invariance (common loadings across grade). (d) Model 3: Scalar invariance (common loadings and intercepts across grade). The chi-square statistic, the Tucker-Lewis Index (*TLI*), the comparative fit index (*CFI*), and the root-mean-square error of approximation (*RMSEA*) were used to assess model fit. The criteria for acceptable model are the *CFI*, *TLI* > 0.90, the *SRMR* < 0.05, and the *RMSEA* < 0.08 (Little, 2013). The chi-square differences between models (*p* > 0.05) and the differences of model fit (∆*CFI* < 0.01) were considered as the indications of invariant measurements (Cheung and Rensvold, 2002; Chen, 2007; Hair et al., 2014).

It should be noted that all variables were standardized before testing the mediation model and moderated mediation model. Moreover, both direct and indirect effects were estimated with 95% confidence interval (CI) based on the bias-corrected percentile method with 1,000 bootstrap samples.

## 3. Results

### 3.1. Common method bias

Harman’s One-Factor Test was used for factor analysis (unrotated exploratory factor analysis) for all items in this study (Podsakoff et al., 2003). The results showed that the eigenvalues of 18 factors were greater than 1, and the variance explained by the first factor was 23%, less than 40%, which indicated that the common method bias did not exist in the present study.

### 3.2. Descriptive statistics and correlation analyses

The means, standard deviations, skewness, and kurtosis (minus 3) of the main variables are presented in Table 1, and the correlation matrix of the main variables are presented in Table 2. The results showed that meaning in life (MIL), school adjustment (SAD), and smartphone addiction (SA) were very approximately normal distribution in the present study. Furthermore, meaning in life was positively correlated with school adjustment (*r* = 0.51, *p* < 0.01) and negatively correlated with smartphone addiction (*r* = −0.50, *p* < 0.01). School adjustment was negatively correlated with smartphone addiction (*r* = −0.58, *p* < 0.01).

**Table 1:** Descriptive statistics of the variables (*n* = 1,076).

Variables	*M* ± *SD*				Skewness	Kurtosis (−3)
	Female freshmen (*n* = 369)	Female sophomores (*n* = 379)	Female juniors (*n* = 328)	Total score (*n* = 1,076)		
1 MIL	44.57 ± 7.66	36.32 ± 7.52	41.28 ± 6.89	40.66 ± 7.63	1.62	2.46
2 SAD	114.92 ± 11.19	97.34 ± 15.07	102.99 ± 13.89	105.09 ± 13.48	1.75	1.78
3 SA	39.89 ± 7.81	51.25 ± 10.40	46.08 ± 8.03	45.78 ± 8.73	1.45	0.36

MIL, meaning in life; SAD, school adaptation; SA, smartphone addiction.

**Table 2:** Inter-correlations of the variables (*n* = 1,076).

Variables	1	2	3
1 MIL	1		
2 SAD	0.51**	1	
3 SA	−0.50**	−0.58**	1

MIL, meaning in life; SAD, school adaptation; SA, smartphone addiction.

** *p* < 0.01.

### 3.3. Testing the mediation model

As shown in Table 3, meaning in life significantly negatively predicted smartphone addiction (*β* = −0.28, *z* = −5.72, *p* < 0.001) and significantly positively predicted school adjustment (*β* = 0.50, *z* = 10.86, *p* < 0.001), and school adjustment significantly negatively predicted smartphone addiction (*β* = −0.43, *z* = −8.81, *p* < 0.001). The mediating effect of school adjustment was −0.21, accounting for 43% of the total effect, and the 95% confidence interval (95% CI: −0.32 to −0.14) excluded 0. The direct effect was −0.28, accounting for 57% of the total effect, and the 95% confidence interval (95% CI: −0.37 to −0.18) excluded 0. The fit indices of the mediation model showed that *χ*^2^(90.384)/*df* (48) = 1.883, *RMSEA* = 0.056 (90% CI: 0.034–0.078), *TLI* = 0.969, *CFI* = 0.978, and *SRMR* = 0.033, which all reached the criteria of good model fit.

**Table 3:** Testing the mediation effect of school adaptation.

Independent variables	Equation 1: SA			Equation 2: SAD			Equation 3: SA		
	*β*	*SE*	*z*	*β*	*SE*	*z*	*β*	*SE*	*z*
MIL	−0.49	0.05	−10.66***	0.50	0.05	10.86***	−0.28	0.05	−5.72***
SAD							−0.43	0.05	−8.81***
*R* ^2^	0.26			0.26			0.40		
*F*	60.60			60.36			75.26		

MIL, meaning in life; SAD, school adaptation; SA, smartphone addiction.

*** *p* < 0.001.

### 3.4. Measurement invariance across grade

As shown in Table 4, the measurement model showed an acceptable fit to the data for female freshmen, *χ*^2^(*df* = 32) = 39.626, *p* < 0.001, *CFI* = 0.962, *SRMR* = 0.037, *TLI* = 0.943, *RMSEA* = 0.064, for female sophomores, *χ^2^* (*df* = 32) = 46.927, *p* < 0.001, *CFI* = 0.941, *SRMR* = 0.041, *TLI* = 0.922, *RMSEA* = 0.073, and for female juniors, *χ*^2^(*df* = 32) = 74.917, *p* < 0.001, *CFI* = 0.944, *SRMR* = 0.039, *TLI* = 0.925, *RMSEA* = 0.062. Thus, the measurement invariance tests can be performed next.

**Table 4:** Measurement invariance tests for freshman, sophomores and juniors.

Model tested	Model fit measures							Model differences			
	*χ^2^*	*df*	*p*	*CFI*	*SRMR*	*TLI*	*RMSEA*	∆*CFI*	∆*χ^2^*	∆*df*	*p*
Separate groups											
Female freshmen	39.626	32	0.000	0.962	0.037	0.943	0.064				
Female sophomores	46.927	32	0.000	0.941	0.041	0.922	0.073				
Female juniors	74.917	32	0.000	0.944	0.039	0.925	0.062				
Model 1: Configural invariance	161.470	96	0.000	0.951	0.036	0.936	0.067				
Model 2: Metric invariance	172.282	110	0.000	0.946	0.040	0.932	0.065	<0.01	10.812	14	0.701
Model 3: Scalar invariance	192.121	124	0.000	0.941	0.043	0.929	0.072	<0.01	19.839	14	0.135

Model 1 (configural invariance), model 2 (metric invariance), and model 3 (scalar invariance) all fit well. The model comparison showed that there was no significant difference between model 2 and model 1 in the fit indices (∆*χ*^2^ = 10.812, ∆*df* = 14, *p* > 0.05, ∆*CFI* < 0.01). Further comparing model 3 with model 2, the result found that there was no significant difference in the fit indices between the two models (∆*χ^2^* = 19.839, ∆*df* = 14, *p* > 0.05, ∆*CFI* < 0.01). The above results indicated that the scalar level of invariance (strong invariance) across grade was established for all measures, which satisfied the requirements of structural model comparison.

### 3.5. Grade differences in direct effect and indirect effect

In order to compare the grade difference of direct effect and indirect effect, the fit indices of the unconstrained structural model (totally free for each group) and the constrained structural model (all the paths equal across groups) were first compared. The results (see Table 5) showed that the fit indices of the unconstrained structural model were good, while those of the constrained structural model were poor. There was a significant difference between the two models (∆*χ^2^* = 25.699, *df* = 6, *p* < 0.001, ∆*CFI* > 0.01).

**Table 5:** Comparison of fit indices between unconstrained structural model and constrained structural model.

Model tested	Model fit measures							Model differences			
	*χ^2^*	*df*	*p*	*CFI*	*SRMR*	*TLI*	*RMSEA*	△*CFI*	△*χ^2^*	△*df*	*p*
Unconstrained structural model	188.039	110	0.000	0.956	0.038	0.933	0.063				
Constrained structural model	213.738	116	0.000	0.896	0.056	0.887	0.085	>0.01	25.699	6	<0.001

Further comparing the differences of path coefficients among female freshmen (group 1), female sophomores (group 2), and female juniors (group 3), the research results (see Table 6) showed that: (a) There was no significant difference between female freshmen, female sophomores, and female juniors in the path coefficient of MIL→SA. (b) There was no significant difference between female freshmen, female sophomores, and female juniors in the path coefficient of MIL→SAD. (c) The path coefficient of SAD→SA in female juniors was not significant. (d) The path coefficient of SAD→SA in female sophomores was significantly larger than that of female freshmen. (e) School adjustment mediated the relationship between meaning in life and smartphone addiction in female freshmen and sophomores. The mediating effect of school adjustment in female freshmen was −0.22 (95% CI: −0.39 to −0.15), and the mediating effect of school adjustment in female sophomores was −0.39 (95% CI: −0.58 to −0.29). (f) School adjustment could not mediate the relationship between meaning in life and smartphone addiction in female juniors.

**Table 6:** The results of grade comparison in path coefficients.

	Pathway 1: MIL → SA			Pathway 2: MIL → SAD			Pathway 3: SAD → SA		
	*β*	*SE*	*Z*	*β*	*SE*	*Z*	*β*	*SE*	*Z*
Group 1: Female freshmen	−0.35	0.11	−3.11***	0.48	0.09	5.39***	−0.45	0.07	−6.53***
Group 2: Female sophomores	−0.37	0.14	−2.65**	0.56	0.08	7.20***	−0.69	0.13	−5.35***
Group 3: Female juniors	−0.25	0.12	−2.21*	0.45	0.13	3.43***	−0.13	0.10	−1.29
Group 1 *VS* Group 2	0.02	0.18	0.13	−0.08	0.15	−0.52	0.24	0.07	3.57***
Group 1 *VS* Group 3	−0.10	0.16	−0.65	0.03	0.16	0.22			
Group 2 *VS* Group 3	−0.12	0.19	−0.64	0.11	0.17	0.66			

MIL, meaning in life; SAD, school adaptation; SA, smartphone addiction.

* *p* < 0.01.

** *p* < 0.01.

*** *p* < 0.001.

## 4. Discussion

This study constructed a moderated mediation model to explore the underlying mechanisms between meaning in life and female college students’ smartphone addiction. Through testing the model, it was not only clear how meaning in life affects female college students’ smartphone addiction (the mediating effect of school adjustment), but also under what conditions meaning in life has a more significant impact on female college students’ smartphone addiction (the moderating effect of grade). The results of this study have theoretical guidance and practical significance for preventing and intervening in female college students’ smartphone addiction.

### 4.1. The mediating effect of school adjustment

Consistent with previous studies (Çevik et al., 2020; Hu et al., 2022), this study found that meaning in life significantly negatively predicted college students’ smartphone addiction. This result is also congruent with meaning therapy theory (Frankl, 1963); that is, the most fundamental drive of human existence is to constantly discover the meaning and purpose of life. Without meaning in life, individuals are likely to fall into a state of emptiness, resulting in some psychological symptoms (Park and Baumeister, 2016; Lin, 2020) and addictive behaviors (Yao et al., 2016; Çevik et al., 2020; Hu et al., 2022). A prior study verified that the higher the level of college students’ life meaning, the less likely they are to have addictive behaviors (Steger and Frazier, 2005). Besides, based on meaning therapy theory, Wang J. J. (2017), Wang X. (2017) found that meaning therapy can significantly interfere with smartphone addiction, further demonstrating that meaning in life is an important factor affecting smartphone addiction. College students may use their smartphones for a number of online activities to escape reality. When college students feel empty and anxious due to a lack of meaning in their lives, they may become addicted to the Internet to escape boredom and release negative emotions, thus leading to smartphone addiction (Hu et al., 2022).

This study further found that school adjustment mediated the relationship between meaning in life and smartphone addiction. This means that a higher level of meaning in life can promote the development of female college students’ school adjustment, in turn reducing female college students’ smartphone addiction; a lower level of meaning in life can hinder the development of college students’ school adjustment, in turn increasing the risk of female college students’ smartphone addiction. In the first path of the mediation process, we found that meaning in life significantly and positively predicted female college students’ school adjustment. This result is congruent with meaning in life theory (Roepke et al., 2014; Glaw et al., 2017; Hooker et al., 2018); that is, a higher level of meaning in life promotes the development of social adjustment. According to the model linking meaning in life to health proposed by Hooker et al. (2018), individuals who have a clear and consistent sense of meaning in life are more aware that their lives are meaningful in their daily interactions with the world, and they tend to better exert self-regulation, feel less daily stress, adopt adaptive coping skills, engage in healthy behaviors, and avoid behaviors that endanger their health. School adjustment is the main component of college students’ social adjustment. Consequently, meaning in life also contributes to college students’ school adjustment. Some empirical studies also support this result. On the one hand, the higher the level of college students’ meaning in life, the less stress, anxiety, and depression they will experience (Park and Baumeister, 2016; Lin, 2020). On the other hand, the higher the level of college students’ meaning in life, the higher their life satisfaction and happiness (McMahan and Renken, 2011; Steger et al., 2011; Lin, 2020). We also found that school adjustment significantly and negatively predicted smartphone addiction in the second path of the mediation process, which is congruent with self-determination theory. The self-determination theory suggests that individuals who satisfy their psychological needs have stronger social adaptability, and their risk of addiction is lower; individuals with frustrated psychological needs may experience less meaning in life, and they are more prone to maladjustment, which may lead them to seek substitutes and compensation in other fields (Deci and Ryan, 2000; Kuzucu and Şimşek, 2013). Smartphones have become essential for college students in their daily lives and studies. Because of its functional characteristics (e.g., collectivity of functions, personalization and customization of content, accessibility, and convenience), smartphones become a tool for college students with school maladjustment to obtain alternative satisfaction and compensation, which easily leads to smartphone addiction (Aker et al., 2017; Boumosleh and Jaalouk, 2017; Kim et al., 2017; Bian et al., 2018; Alinejad et al., 2022). Therefore, based on empirical and theoretical evidence, the above relationship between meaning in life, school adjustment, and smartphone addiction could be well comprehended.

In summary, the present study provided evidence that meaning in life could not only directly affect female college students’ smartphone addiction, but also indirectly affect female college students’ smartphone addiction through school adjustment. According to these results, regardless of grade differences, we can understand that the stronger the meaning in life of female college students, the stronger their overall school adjustment, and the less likely they are to be addicted to smartphones. On the contrary, female college students who lack meaning in life are prone to school adjustment problems. In order to relieve psychological pressure and negative emotions caused by school maladjustment and obtain alternative satisfaction and compensation, they are easily addicted to the “virtual world” of smartphones (Song and Zhao, 2021).

### 4.2. The moderating effect of grade

By conducting multiple group analysis, it was found that school adjustment had a partial mediating effect between meaning in life and smartphone addiction for female freshmen and sophomores, while for female juniors, the mediating effect of school adjustment was not significant. The comparison of the grade difference of the paths revealed that there was no significant grade difference in the path coefficients of MIL→SA and MIL→SAD, but there was a significant grade difference in the path coefficient of SAD→SA. The path coefficient of SAD→SA in female sophomores was significantly larger than that of female freshmen. According to identity-based motivation theory (Oyserman, 2009a,b), people will behave in a way that is consistent with the identity initiated in a certain situation. But this kind of “identity” is not stable, and it will change with the change of external environment. In different grades, college students need to face different external environmental challenges. Adapting to the university quickly is a major challenge for freshmen (Lu, 2015; Zhang Y. L. et al., 2017; Zhang Y. et al., 2017). After entering the university, freshmen are full of passion and expectation for the upcoming university life, and they actively adjust themselves to adapt to the university life (Lu, 2015). Besides, college teachers pay more attention to their study, life, and mental health (Zhang Y. L. et al., 2017; Zhang Y. et al., 2017). All of these help them to form their possible selves which are in line with the school environment requirements of freshman year, and then promote the formation of their healthy identities. Thus, female freshmen’s meaning in life is the highest compared with other grades in this study (see Table 1), which is consistent with the results of previous studies (Zhang and Zhou, 2020; Xie, 2021). The high level of meaning in life helps them to develop good school adjustment, in return reducing the possibility of their smartphone addiction. Sophomores formally enter the stage of autonomous learning, and their learning pressure in specialized courses has further increased (Gahagan and Stuart Hunter, 2008; Sanchez-Leguelinel, 2008). They do not feel so fresh about college life any more, and they start to lose interest in it (Richmond and Lemons, 1985; Lu, 2015; Zhang Y. L. et al., 2017; Zhang Y. et al., 2017). Besides, after the students enter the sophomore year, the degree of attention paid by teachers to them is reduced, which easily leads to many students’ depressions and problems in their study and life (Lu, 2015; Zhang Y. L. et al., 2017; Zhang Y. et al., 2017). The above phenomenon among sophomores is called the “sophomore slump” by scholars. Obviously, sophomores need to face very severe challenges. Under the influence of the above-mentioned internal and external unfavorable factors, sophomores may form negative possible selves, which may conflict with the school environment requirements (developing autonomous learning ability) of sophomore year, leading to their “identity crisis” (Li and Liu, 2010). As a result, female sophomores’ meaning in life is seriously lower than that of their freshman year in this study (see Table 1), which is consistent with the results of previous studies (Zhang and Zhou, 2020; Xie, 2021). The low level of meaning in life may lead to their serious school maladjustment, further leading to serious smartphone addiction. Consequently, it can be easily understood that compared with freshmen, sophomores are more prone to smartphone addiction due to school maladjustment. Compared with freshmen and sophomores, entering a higher school and employment are the main stressors for juniors (Lu, 2015). Under the influence of these pressures, juniors have to make great efforts to cope with the challenges of entering a higher school or employment. They began to reflect on their sophomore life and study, think about their life direction, and adjust their behaviors to truly adapt to the university (Zeng and He, 2015). In short, juniors are developing in the direction of forming a healthy identity. Therefore, consistent with the results of previous studies (Zhang and Zhou, 2020; Xie, 2021), female juniors’ meaning in life is higher compared with female sophomores in this study (see Table 1), which contributes to the development of their school adjustment. In addition, since entering a higher school and employment have replaced school adjustment as the main stressors of juniors, the pressures of entering a higher school and employment are probably the inducing factors that leads to female junior students’ smartphone addiction, while the pressure of school adjustment is not. Thus, it is easy to understand meaning in life could not affect female juniors’ smartphone addiction through school adjustment.

To sum up, the relationship between meaning in life, school adjustment, and smartphone addiction was moderated by grade. For female freshmen, the high level of meaning in life promotes the development of their school adjustment, thus reducing their risk of smartphone addiction. For female sophomores, the low level of meaning in life may lead to their school maladjustment, thus increasing their risk of smartphone addiction. For female juniors, meaning in life could not mediated the relationship between meaning in life and smartphone addiction.

## 5. Conclusion

To sum up, this study explored the relationship between meaning in life, school adjustment, and smartphone addiction and the moderating effect of grade on the above relationship among female college students, which can help us to further understand the mechanism of female college students’ smartphone addiction. Firstly, we found that meaning in life could influence female college students’ smartphone addiction through school adjustment without considering grade differences. Then, this study further revealed that meaning in life could affect smartphone addiction through school adjustment for female freshmen and sophomores, but the mediating effect of school adjustment was not significant for female juniors. Moreover, the influence of school adjustment on female sophomores’ smartphone addiction was significantly stronger than that of female freshmen. These results imply that while college teachers pay attention to freshmen’s school adjustment, sophomores’ school adjustment can never be ignored. These findings provide ideas for the prevention or intervention of female college students’ smartphone addiction from the perspective of grade difference.

## 6. Limitations, future directions, and implications

Some limitations in this study merit addressing in future study. First, all participants in this study are Chinese female college students. Future study is needed to examine these findings among female college students in other countries and cultures. Second, although the hypotheses of this study were based on some theories, the research design of this study was cross-sectional. We can conduct longitudinal research to examine the causal relationship between meaning in life, school adjustment, and smartphone addiction among female college students in the future. Third, this study only examined the influence of individual factors on female college students’ smartphone addiction. However, the development of individuals is influenced by many different systems (Bronfenbrenner, 1979). In the future, it is important to further examine how family factors, school factors, peer factors, and individual factors systematically influence female college students’ smartphone addiction.

Although there are some limitations in this study, the results have significant implications. First, from the perspective of theoretical significance, this study deepened the previous studies on the mechanism of female college students’ smartphone addiction by examining the relationship between meaning in life, school adjustment, and smartphone addiction and the moderating effect of grade on the relationship, and it revealed the mediating effect of school adjustment between meaning in life and smartphone addiction and the differences of the mediating effect in different grades. These findings can further promote the understanding of the potential impacts of meaning in life on smartphone addiction. Second, from the perspective of practical significance, the findings of this study can provide targeted guidance for the prevention and intervention of female college students’ smartphone addiction. The findings suggested that we should consider the grade differences when preventing or intervening in female college students’ smartphone addiction. For female freshmen, we can consider preventing their smartphone addiction by enhancing their meaning in life and school adaptability. For female sophomores, we can consider intervening in their smartphone addiction by enhancing their meaning in life and school adaptability. For juniors, we can focus on meaning in life and adopt meaningful therapy to intervene in their smartphone addiction. For example, the meaning analysis method in meaningful therapy can be used to help female college students analyze the meaning of life to enhance their meaning in life. For female freshmen and sophomores, group psychological counseling can be carried out to improve their school adaptability.

## Data availability statement

The raw data supporting the conclusions of this article will be made available by the authors, without undue reservation.

## Ethics statement

The studies involving human participants were reviewed and approved by Academic Committee of Shandong Women’s University. Written informed consent to participate in this study was provided by the participants.

## Author contributions

HZ: methodology, formal analysis, and writing—original draft. SR-G: revised, validation, and supervision. MF: writing—review and editing and investigation. TS: translation and polishing. All authors contributed to the article and approved the submitted version.

## Conflict of interest

The authors declare that the research was conducted in the absence of any commercial or financial relationships that could be construed as a potential conflict of interest.

## Publisher’s note

All claims expressed in this article are solely those of the authors and do not necessarily represent those of their affiliated organizations, or those of the publisher, the editors and the reviewers. Any product that may be evaluated in this article, or claim that may be made by its manufacturer, is not guaranteed or endorsed by the publisher.
